# Characterization of Staphylococcus argenteus in Christchurch, New Zealand, and comparison to global strains

**DOI:** 10.1099/acmi.0.000916.v4

**Published:** 2025-07-16

**Authors:** Trevor Anderson, Hui Wang, Michael Harrington, Julia C. Howard, Erik Otte

**Affiliations:** 1 Canterbury Health Laboratories, Christchurch Hospital, Health New Zealand, Christchurch, Canterbury, New Zealand

**Keywords:** antimicrobial resistance, phylogenetics, *Staphylococcus argenteus*, *Staphylococcus aureus*, staphyloxanthin, virulence factors, whole-genome sequencing

## Abstract

*Staphylococcus argenteus* (SARG) was discovered in 2009 as part of the *Staphylococcus aureus* (SAUR) complex and has been documented from various locations worldwide. In this article, we describe the genomic features of five strains of SARG found in Christchurch, New Zealand. Isolates were first detected in 2019 using MALDI-TOF identification, and their identities were confirmed using whole-genome sequencing. Genomic features, including antimicrobial resistance markers and virulence factors, were compared with other SARG sequences in the NCBI GenBank and well-characterized features in SAUR. Four isolates belonged to ST2250 and one isolate to ST2793. Phylogenetic analysis based on core genome analysis revealed that all five isolates were phylogenetically distinct, with four isolates clustering in the ST2250 clade. Three isolates contained staphylococcal cassette chromosome *mec* (SCC*mec*) type IV 2Bc, harbouring the *mecA* gene conferring resistance to beta-lactam antibiotics. All five strains shared many of the virulence genes found in the global SARG and SAUR isolates; however, no TSST-1 or PVL pathogenic genes were detected. This publication contributes additional data on global occurrences and genomic features of SARG.

**Impact Statement**

This article reports the occurrence of *Staphylococcus argenteus* (SARG) from five routinely collected clinical samples as part of routine clinical care in New Zealand, further indicating its global spread. Whole-genome sequencing revealed traditional *Staphylococcus aureus* virulence factors and resistance genes adding to the evidence regarding the pathogenic potential of SARG.

## Data Summary

All raw MiSeq sequence data and assemblies have been added to GenBank, accession numbers: PRJNA957538–SAMN34257150 (19CHL-88664D), 20CHL-2391H (SAMN34257151), 20CHL-9983R (SAMN34257152), 21CHL-5842I (SAMN34257153) and 22CHL-3048L (SAMN34257154).

## Introduction

*Staphylococcus argenteus* (SARG) is a member of the *Staphylococcus aureus* (SAUR) complex and was first discovered in tropical northern Australia as a unique lineage within SAUR using MLST and *spa* typing. Initially referred to as clonal complex 75, SARG has been subsequently found in other populations around the world [1,3]. A recent article by Witteveen *et al*. [4] has shown evidence of SARG circulating in the Netherlands since at least 2008 [4]. Additionally, isolates have been described in animals in small numbers: from a cow in Malaysia, a pig in China, a gorilla in Gabon [5], a dog in the Netherlands [6] and in retail chicken samples in China and Japan, as well as in slaughterhouses in Japan [7].

There are no phenotypic features that can reliably identify SARG from SAUR other than the lack of staphyloxanthin (golden pigment) production [4,8]. Genomic analysis has found that SARG lacks the carotenoid biosynthetic operon *crtOPQMN*. MALDI-TOF MS may differentiate SARG from SAUR [9], but molecular assays such as *nuc* gene PCR [10] or DNA sequencing of various genes are required for confirmation [11,13]. SARG strains encoding the *mecA* gene within the SCC*mec* region may also be falsely identified as methicillin-resistant *Staphylococcus aureus* (MRSA) when screening samples using phenotypic testing. Subsequently, SARG isolates are likely commonly misidentified, partly explaining why they are rarely detected in the clinical laboratory. This was demonstrated by a study from Thailand that retrospectively examined 246 consecutively collected SAUR isolates and found that 4.1% have matching MLST *arC* and *pta* allele profiles associated with SARG as determined by phylogenetic clustering [2].

SARG has been isolated from a range of clinical sites. Although it was initially considered to be less pathogenic than SAUR, there are increasing reports of serious invasive disease including bacteraemia and bone and joint infections caused by the organism [14,15]. It has been suggested that the lower pathogenicity of SARG could be due to a lack of staphyloxanthin production, which protects against oxidative stress [12]. SARG strains have been described as harbouring pathogenic genes such as Panton–Valentine leukocidin (PVL) and toxic shock syndrome toxin-1 (TSST-1) [16,18], and an invasive infection study from Thailand found that SARG has similar rates of morbidity and mortality as methicillin-susceptible SAUR [16].

Plasmids play a crucial role in bacterial evolution and often carry resistance genes, virulence factors and metabolic capabilities, such as heavy metal resistance. Horizontal gene transfer is an important mechanism for plasmid exchange and has been documented between *Staphylococcus* spp. [19,21].

In this article, we describe the genomic features of five SARG isolates from New Zealand (NZ), comparing genomic characteristics including antimicrobial resistance genes and pathogenic markers, with global SARG isolates available in the NCBI GenBank. SARG is part of the SAUR complex and closely related to SAUR. Therefore, we also compared key pathogenic markers and other genomic features with those of this well-characterized pathogen.

## Methods

This study involved five identified routinely collected clinical isolates of SARG received between 2019 and 2022, which underwent whole genome sequencing (WGS) to confirm their identification. As per usual laboratory processes for routine MRSA screening, isolates from swab samples (nose, groin and other skin sites) were recovered from SAUR selective enrichment broth, which was sub-cultured onto BBL™ CHROMagar™ MRSA II agar (Fort Richards, Auckland, NZ). All media were incubated at 35 °C for 24 h in an ambient atmosphere. Pink colour colonies from chromogenic agar or colonies resembling SAUR from 5% sheep blood agar (SBA) were identified as SAUR/SARG using Bruker Microflex LT (Bruker Daltonics, Bremen, Germany). A blood culture isolate was plated from a BD BACTEC Plus aerobic bottle inoculated onto 5% SBA. MALDI-TOF MS was performed on pure culture of the isolate by inoculation onto the MALDI target plate using a wooden applicator, followed by the application of 1 µl formic acid, which was air dried before applying 1 µl α-cyano-4-hydroxycinnamic acid matrix; results were analysed with a database containing 6,903 Mass Spectral Profiles (MSP). Isolates were sub-cultured onto 5% SBA and stored at −80 °C in cryo-preserved vials.

### Antimicrobial susceptibility testing

Isolates were tested using European Committee on Antimicrobial Susceptibility Testing (EUCAST) guidelines and categorized as susceptible (S), susceptible increased exposure (I) or resistant (R) based on v11.0 breakpoints. One isolate was non-viable from storage, but initial sensitivity results using the BD Phoenix PMIC-84 panel were available. Clindamycin-inducible *erm*-mediated resistance was screened using the D-zone agar diffusion test with erythromycin [22].

### Molecular analysis

Nucleic acid was extracted using the QIAamp UPC pathogen mini kit (Qiagen, Auckland, NZ) using mechanical pre-lysis and spin protocol as recommended in the handbook (v04/2021). DNA was quantitated for WGS using Qubit fluorometer (Thermo Fisher Scientific) with the dsDNA broad range kit. WGS DNA library preparation was performed using the Illumina DNA Flex library kit (Illumina, Australia). Libraries were sequenced using MiSeq (Illumina) with micro flow-cell, reagent kit v2 (300 cycles) according to the manufacturer’s instructions. Bioinformatic analysis was performed with the following software with default settings, unless stated otherwise. FASTQ paired-end reads were quality trimmed and assembled into contiguous (contigs) sequences using Shovill v0.9.0 (https://github.com/tseemann/shovill). Genome assembly quality and accuracy were checked using CheckM2 [23]. Genomic analysis was performed with the following software: Kraken 2 [24], AMRFinder [25], MLST (https://github.com/tseemann/mlst) and Abricate (https://github.com/tseemann/abricate) with MEGARes database (6,635, 3 December 2024). SCC*mec* typing and plasmid (PlasmidFinder) typing were performed via the Center for Genomic Epidemiology online server http://www.genomicepidemiology.org/services/ (accessed 14 February 2022). Additionally, 227 global SARG sequences from RefSeq (NCBI) were downloaded on 29 November 2024 for comparative analysis. Dereplicator software (https://github.com/rrwick/Assembly-Dereplicator) was used to filter 52 redundant sequences based on a 0.001 distance score. One hundred and eighty FASTA sequence files were annotated using Prokka [26], and the GFF3 files were then analysed by the Panaroo program v1.5.1 [27] to create a core genome. Phylogenetic analysis was performed on the core genome SNP alignment with Gubbins v3.3.1 [28] recombination filtering and IQ-TREE v2.3.6 [29] tree construction using maximum-likelihood modelling with 1,000 bootstrap calculations. Plasmid contigs were reoriented around the rep gene using dnaapler v1.1.0 (https://github.com/gbouras13/dnaapler) and annotated using bakta v1.1.10 software [30]. The annotated sequences were then aligned using the MAFFT plugin in Geneious Prime (2025.0.2). Annotated genomes were viewed for gene synteny using Geneious Prime.

Whole-genome data from this publication can be found in the NCBI GenBank under BioProject PRJNA957538. BioSample numbers are as follows: SAMN34257150 (19CHL-88664D), SAMN34257151 (20CHL-2391H), SAMN34257152 (20CHL-9983R), SAMN34257153 (21CHL-5842I) and SAMN34257154 (22CHL-3048L).

## Results

Four SARG were isolated from skin sites and one from blood culture. Isolates grown on 5% SBA typically lacked the characteristic golden pigment (staphyloxanthin), which is normally present in SAUR colonies. MALDI-TOF MS profiles failed to differentiate SARG from SAUR with a best match score of ≥1.9 but highlighted potential isolates for further analysis. Identification as SARG was confirmed using WGS with Kraken 2 and blastn (https://blast.ncbi.nlm.nih.gov/) of the thermonuclease (*nuc*) gene. The analysis of the annotated sequences found no complete carotenoid biosynthetic operon (*crtP*, *crtQ*, *crtM* or *crtN* genes), which is consistent with the phenotypic appearance. blastn analysis of the *nuc* gene, which is ~675 bp, found matching nt identities of 98–100% to other SARG sequences but only 93% match with SAUR and *Staphylococcus schweitzeri*.

The FASTQ files were assembled using the Shovill pipeline that uses the Spades assembler [31], and genome assembly checkm2 metrics are available in Table 1.

**Table 1.** NZ SARG genomic assembly metrics

Sample	Contig	Genome size (Mbp)	N50 (bp)	GC (mol%)	CDS	Kraken 2 ID
19CHL-8664D	34	2.8	297,335	32.29	2,667	SARG
20CHL-2391H	38	2.76	388,839	32.26	2,597	SARG
20CHL-9983R	29	2.8	349,638	32.27	2,661	SARG
21CHL-5842I	22	2.72	487,332	32.31	2,566	SARG
22CHL-3048L	91	2.75	140,563	32.26	2,569	SARG

### Antimicrobial susceptibility

The NZ SARG isolates were susceptible to a wide range of antimicrobials with notable resistance to penicillin and flucloxacillin. The whole-genome analysis also detected *blaZ* and *mecA* genes which encode beta-lactam resistance and *fosB* encoding fosfomycin resistance as seen in Table 2. No EUCAST interpretative guidelines are available for fosfomycin disc susceptibility testing, but the presence of *fosB* has been described in SAUR as a mechanism of resistance [32]. The three methicillin-resistant isolates had SCC*mec* mobile genetic element type IVc (2B) containing IS6 family transposase (IS431mec) and *mecA*, *mecI* and *mecR* genes. The analysis of 175 global strains from GenBank showed that *blaZ* is the predominant mechanism for penicillin resistance, present in 111 (63.4%) isolates (Table 3). The other beta-lactam resistance mechanism detected was altered penicillin-binding protein (PBP) 2a encoded by the *mecA* gene found in 58 (33.1%) isolates. Other significant resistance markers included *fosB* in 152 (86.9%) isolates, *aph*(3′)-IIIa (aminoglycoside resistance) in 55 (31.4%) and *dfrG* (trimethoprim resistance) in 41 (23.4%) (see Table 3). The five NZ isolates were relatively susceptible with no macrolide or lincosamide (inducible or constitutive), fluoroquinolone or tetracycline resistance by phenotypic or genotypic testing. Other resistance mechanisms detected included multidrug and toxic compound extrusion (MATE) and major facilitator superfamily (MFS) efflux pumps, such as tetracycline resistance (TET38).

**Table 2.** NZ SARG phenotypic and genotypic susceptibility profile

Antimicrobial (µg)	19CHL-8664D	20CHL-9983R	20CHL-2391H	21CHL-5842I	22CHL-3048L*
Penicillin (10U)	R (10) [*blaZ*]	R (10) (*blaZ*)	S (38)	S (38)	R (*blaZI*)
Flucloxacillin (1)	R (11) [*mecA*]	R (12) (*mecA*)	S (22)	S (22)	R (*mecA*)
Gentamicin (10)	S (22)	S (21)	S (22)	S (23)	S
Erythromycin (15)	S (23)	S (25)	S (26)	S (24)	S
Clindamycin (2)	S (23)	S (24)	S (28)	S (25)	S
Co-trimoxazole (25)	S (19)	S (17)	S (27)	S (30)	S
Tetracycline (30)	S (30)	S (27)	S (30)	S (27)	S
Fosfomycin (200)	(20) (*fosB*)	(25) (*fosB*)	(22) (*fosB*)	(20) (*fosB*)	Not tested
Norfloxacin (10)†	S (23)	S (24)	S (25)	S (24)	S
Clindamycin induction	Not detected	Not detected	Not detected	Not detected	Not detected
Trimethoprim	Not tested (*dfrG*)	Not tested (*dfrG*)	Not tested	Not tested	Not tested (*dfrG*)

EUCAST classification (disc zone size in millimetre) (resistance gene). Classification: R, resistant; S, susceptible. Interpretations based on EUCAST SAUR. *fosB*, FosB1/*FosB3* family fosfomycin resistance (bacillithiol transferase); *dfrG*, trimethoprim-resistant dihydrofolate reductase; *blaZ*, penicillin-hydrolysing class A beta-lactamase; *mecA*, PBP 2 a family beta-lactam-resistant peptidoglycan transpeptidase.

*Isolate was tested using Phoenix (BD, Auckland, NZ); therefore, no zone sizes are available.

†Norfloxacin screening for fluoroquinolone class.

**Table 3.** Comparison of NZ and global SARG resistance markers

Antimicrobial class	Gene	NZ (5)	Global (175)
Aminoglycoside	*ant(4′)-Ia*	0	2
	*aph(2″)-Ih*	0	1
	*aph(3′)-IIIa*	0	55
Beta-lactam	*blaPC1*	0	2
	*blaZ*	3	111
	*mecA*	3	58
Bleomycin	*bleO*	0	2
Fosfomycin	*fosB*	4	152
Fusidic acid	*fusB*	0	1
Lincomycin-Clindamycin	*lnu(A*)	0	1
Macrolide	*erm(C*)	0	2
Macrolide-Streptogramin B	*msr(A*)	0	1
Tetracycline	*tet(38)*	5	175
	*tet(K*)	0	8
	*tet(L*)	0	54
Trimethoprim	*dfrC*	0	1
	*dfrG*	3	41

### Virulence factors

**Fig. 1.**
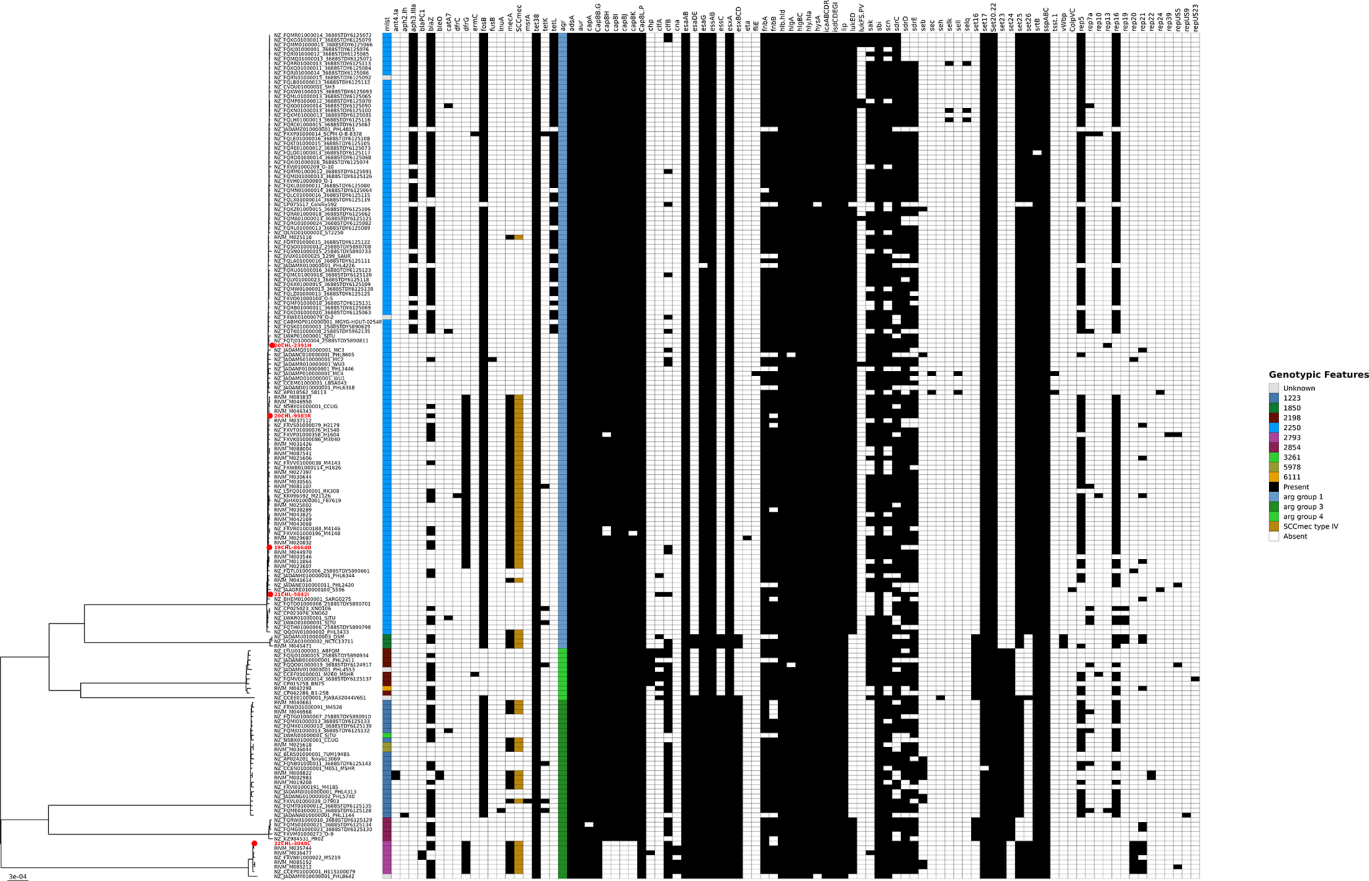
Phylogenetic tree with mapped genomic features. The maximum-likelihood phylogenetic tree from Fig. 3 with genomic features such as MLST, antimicrobial and virulence factors. Data was generated using Abricate software with the MEGARes and VFDB databases, along with PlasmidFinder, SCCmecFinder and AMRfinder.

The analysis of SARG virulence factor profiles revealed that many of the same virulence factors present in SAUR were also found in the NZ SARG isolates, summarized in Table 4. Most virulence factors were common to all isolates with variations such as ESAT-6 secretion system (*esaC*), ESAT-6 cell membrane secretion system (*esxB*), staphylokinase (*sak*), *Staphylococcus* complement inhibitor (*scn*), serine-aspartate repeat-containing proteins (*srdC*/*D*/*E*) and von Willebrand factor-binding protein (*vWbp*). Isolate 22CHL-3048L was missing genes encoding the capsular polysaccharide cap8H-K and clustered with seven global isolates also without these cap8 genes (see Fig. 1). Comparison of virulence factors of 175 global strains from the GenBank showed that they shared the same virulence factors, notably those responsible for capsular production, haemolysin, toxin secretion and fibrinogen factors (see Table 5).

**Table 4.** NZ SARG virulence factors

Gene	Virulence factor	19CHL-8664D	20CHL-9983R	20CHL-2391H	21CHL-5842I	22CHL-3048L
*adsA*	Adenosine synthase A	+	+	+	+	+
*aur*	Aureolysin	+	+	+	+	+
*cap8*(A-P)	Capsular polysaccharide	+	+	+	+	(+)
*clf* (A/B)	Clumping factor	+/+	+/+	+/+	+/+	+/+
*clpP*	Cytoplasmic protease	+	+	+	+	+
*ebp*	Fibrinogen-binding protein	+	+	+	+	+
*esa*(A/B/C)	Type VII protein secretion system	+/+/−	+/+/−	+/+/−	+/+/−	+/+/+
*ess*(A/B/C)	Type VII protein secretion system	+/+/+	+/+/+	+/+/+	+/+/+	+/+/+
*esx*(A/B)	Cell membrane protein	+/−	+/−	+/−	+/−	+/+
*fnb*(A/B)	Fibronectin (Fn)-binding protein	+/+	+/+	+/+	+/+	+/+
*geh*	Glycerol ester hydrolase	+	+	+	+	+
*hlg*(A/B/C)	Gamma haemolysin	+/+/+	+/+/+	+/+/+	+/+/+	+/+/+
*hly/hla*	Alpha haemolysin	+	+	+	+	+
*hysA*	Hyaluronate lyase	+	+	+	+	+
*ica*(A-D/R)	Intercellular adhesion genes	+/+	+/+	+/+	+/+	+/+
*isd*(A-G)	Iron-regulated surface proteins	+	+	+	+	+
*lip*	Lipase	+	+	+	+	+
*luk* (E/D)	Haemolysin	+/−	+/−	+/−	+/−	+/−
*sak*	Staphylokinase	+	+	+	−	−
*sbi*	IgG-binding protein	+	+	+	+	+
*scn*	Staphylococcus complement inhibitor	+	+	+	−	+
*sdr* (C-E)	Serine-aspartate repeat-containing protein	+/−/−	+/+/+	+/+/+	−/+/+	+/+/+
*spa*	Staphylococcal protein A	+	+	+	+	+
*ssp* (A/B/C)	Serine protease	+/+/+	+/+/+	+/+/+	+/+/+	+/+/+
*vWbp*	von Willebrand factor-binding protein	+	+	+	+	−

+, presence; −, absence.

**Fig. 2.**
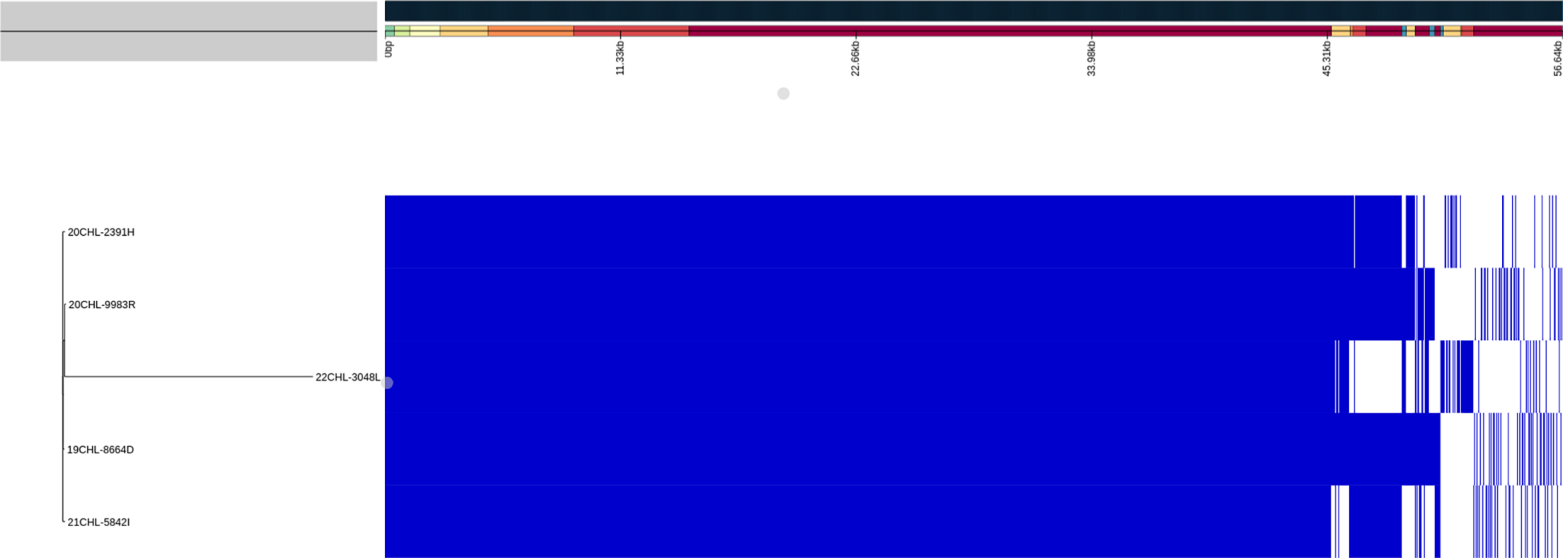
Genome variation of NZ SARG. This figure shows the Panaroo-constructed pangenome with the core genome containing 81.3% gene content. The accessory genome includes unidentified coding regions, pathogenic factors and mobile genetic elements such as plasmids and SCC*mec*.

**Fig. 3.**
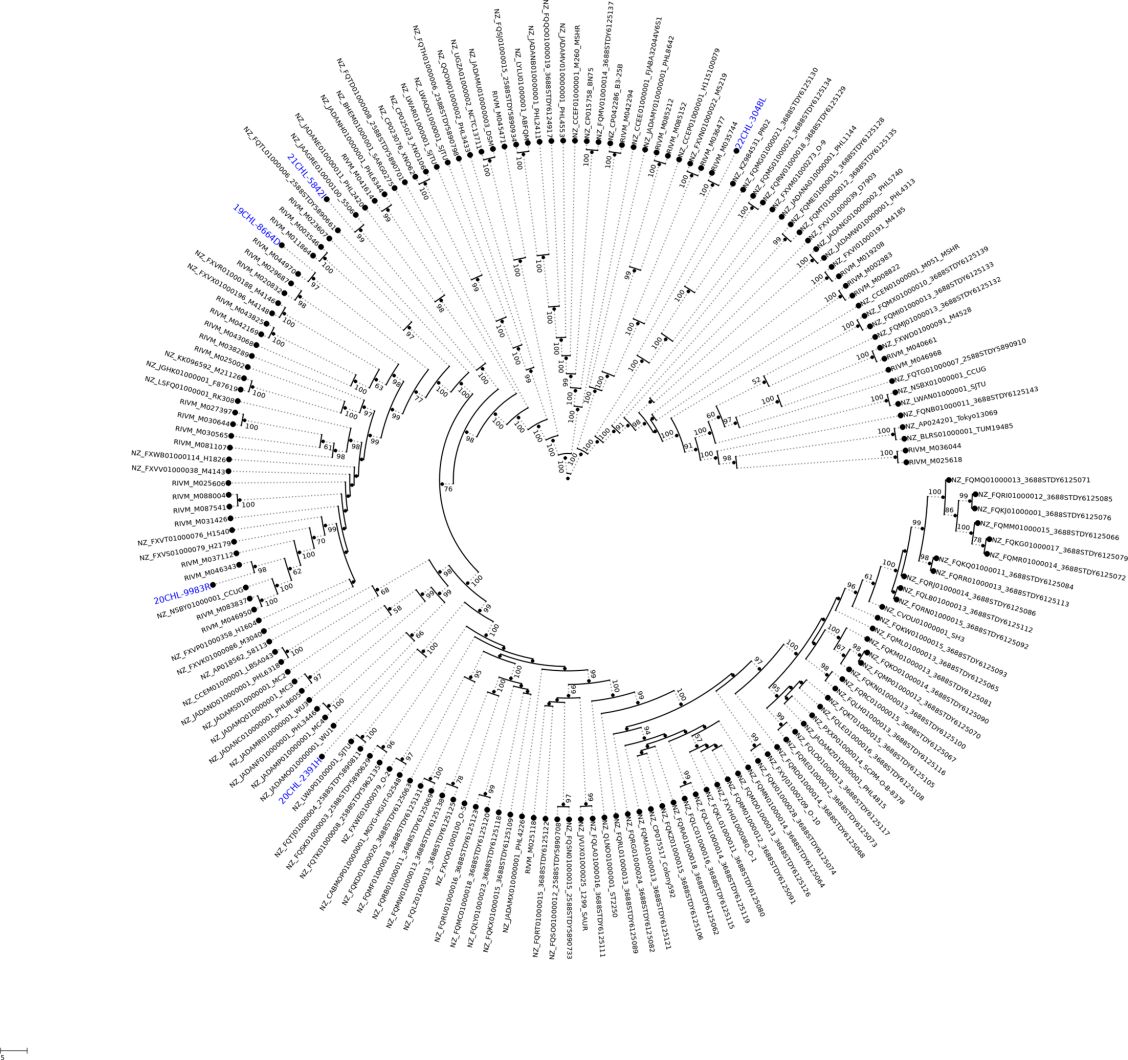
Phylogenetic comparison of NZ and global SARG strains. The distribution of global SARG strains, with NZ strains highlighted, is shown on a mid-point rooted maximum-likelihood phylogenetic tree, created by IQ-TREE (1,000 bootstrap replicates) from the Panaroo core gene SNP alignment. The circular format clearly shows the tree structure and strong branch support values.

**Table 5.** Comparison of NZ and global SARG virulence factors

Pathogenic function	Gene	NZ (5)	Global (175)
Adhesion and biofilm	*clfA*	1	9
Adhesion and biofilm	*clfB*	3	56
Adhesion and biofilm	*cna*	0	5
Adhesion and biofilm	*fnbA*	5	161
Adhesion and biofilm	*fnbB*	3	154
Adhesion and biofilm	*icaA/B/C/D/R*	5	175
Adhesion and biofilm	*sdrC*	5	142
Adhesion and biofilm	*sdrD*	4	141
Adhesion and biofilm	*sdrE*	4	138
Capsule biosynthesis	*cap8A*	5	174
	*cap8B-G*, *cap8L-P*	5	175
	*cap8H*	4	165
	*cap8I/J*	4	168
	*cap8K*	4	167
Haemolysin	*hlb/d*	5	175
Haemolysin	*hlgA*	5	173
Haemolysin	*hlgB/C*	5	175
Immune evasion	*adsA*	5	175
Immune evasion	*chp*	5	175
Immune evasion	*sak*	3	108
Immune evasion	*sbi*	5	175
Immune evasion	*scn*	4	140
Iron acquisition	*isdC/D/E/G/I*	5	175
Protease	*aur*	5	175
Protease	*lip*	5	175
Protease	*sspA/B/C*	5	175
Secretion system	*esaA/B*	5	175
Secretion system	*esaD/E*	1	41
Secretion system	*esaG*	5	175
Secretion system	*essA/B*	5	175
Secretion system	*essC*	1	41
Secretion system	*esxA*	5	175
Secretion system	*esxB/C/D*	1	41
Tissue-degrading enzyme	*hysA*	5	173
Toxin	*eta*	0	1
Toxin	*lukE/D*	5	134
Toxin	*lukF/S-PV*	0	7
Toxin	*seb*	0	11
Toxin	*sec*	0	2
Toxin	*set16*	0	19
Toxin	*set17*	5	174
Toxin	*set20/22*	5	175
Toxin	*tsst-1*	0	2

### Plasmid analysis

Plasmid typing was performed by PlasmidFinder tool, and representative plasmid sequence(s) were downloaded from https://www.ccb.uni-saarland.de/plsdb [33]. SARG penicillin-resistant isolates 19CHL-8664D and 20CHL-9983R contained replication loci (*rep*) types rep16_1_CDS8(pSAS) and rep5a_1_repSAP001(pN315), and isolate 22CHL-3048L contained rep20_3_rep(pTW20). *Rep16* (size range 20,622 to 21,326 bp), *rep20* (size range 20,403 to 33,660 bp) and *rep5a* (size range 19,758 to 59,392 bp) contain the *blaZ*, *blaI* and *blaR1* genes. Penicillin-susceptible isolate 20CHL-2391H had *rep13* type (size range 2,779 to 3,145 bp) with no *blaZ* gene, and isolate 21CHL-5842I had no rep types detected. Analysis of the global strains found that 144 (82.3%) had a replicon locus detected, with the most common types being 119 (68%) rep5 and 127 (72.6%) rep16, with 118 (67.4%) isolates having both replicon types. The same replicon types found in SARG are also in SAUR, indicating a potential exchange of mobile genetic elements [34]. Plasmids rep5a and rep20 also contain the *cadD* gene, which codes for the cadmium efflux system and high-level resistance to this heavy metal [35]. The *blaZ* plasmids for isolates 19CHL-8664D (20,755 bp contig) and 20CHL-9983R (20,736 bp contig) were aligned against plasmid database (PLSDB) (https://ccb-microbe.cs.uni-saarland.de/plsdb2025) sequences and found similar *blaZ* containing plasmids around 20 kb with rep_cluster_1733 and rep_cluster_2214. The 19CHL-8664D and 20CHL-9983R plasmids showed near identical gene synteny, except for 19CHL-8664D that had a 111 bp insert in the *nifR* gene. Isolate 22CHL-3084L also had a *blaZ* gene-containing plasmid (20,108 bp contig), which had 99.9% blastn alignment to plasmid CP096113.1 (25,332 bp) with PLSDB rep_cluster_1215. The 22CHL-3084L plasmid fragment has a 5,225 bp region missing that contains hypothetical proteins, transposons and origin of transfer (*oriT*). Apart from this missing region, the plasmid shows identical gene synteny to the CP096113.1 plasmid. Isolate 20CHL-2391H plasmid fragment (4,788 bp) showed partial alignment on blastn (1,898/1,953 bp) to *Staphylococcus warneri* p12 plasmid (2,140 bp) (AB125342.1) and contains efflux gene *qacC*.

### Molecular typing and phylogenetic analysis

The NZ isolates were sequence typed as ST2250 (*n*=4) and ST2793 (*n*=1), with ST2250 being the most common sequence type (ST) group among the global SARG strains. The analysis of the 175 global strains showed the following ST types: 122 (69.7%) ST2250, 22 (12.6%) ST1223, 8 (4.6%) ST2198, 6 (3.4%) ST2793, 5 (2.9%) ST2854, 3 (1.7%) ST1850, 2 (1.1%) ST5978, 1 (0.6%) ST3261, 1 (0.6%) ST6111 and 6 (3.4%) unknown MLSTs, which have not been uploaded to the PubMLST database.

Pangenome of the NZ isolates constructed using the Panaroo program identified 2,830 gene families, with the core genome containing 2,274 (81.3%) at 95–100% identity. Although four isolates share the same MLST profile, they are genetically distinct based on core genome analysis and accessory gene content (see Fig. 2). The accessory genome contained 556 CDSs that consisted mainly of 388 (69.8%) hypothetical proteins. Additionally, the accessory genome included SCC*mec* elements such as insertion sequences, recombinases, heavy metal resistance (e.g. *cadC* for cadmium resistance) and antibiotic resistance (e.g. *mecA*). Other notable genes found included accessory genes and plasmid-associated genes, such as phage tail proteins, phage major capsid protein and plasmid recombination enzymes. Additionally, a Panaroo pangenome was constructed including 175 global strains. The pangenome identified 3,684 gene families across all 180 strains, with the core genome containing 2,196 (61.1%) genes at 95–100% identity. The maximum-likelihood phylogenetic tree strongly supports the presence of six to seven clades, with the NZ SARG strains distributed across two clades (see Figs 1 and 3). The MLST types align within these clades with a few exceptions. The largest clade, which is predominantly ST2250, contains four of the NZ isolates. Within this ST2250 clade, distinct genomic patterns define various subclades. For example, one subclade includes *aph*(*3′*)*-IIIa* (aminoglycoside resistance), *blaZ* (beta-lactam resistance), *tetL* (tetracycline resistance) genes, *sak* (staphylokinase) and replicon types rep5a and rep16. Another subclade within the ST2250 clade contains strains carrying *dfrG* (trimethoprim resistance) and SCC*mec* (*mecA*, beta-lactam resistance) and the same replicon types (rep5a and rep16). Another notable clade contains the ST2793 strains, which contain isolates lacking some of their *cap8* genes, *dfrG* gene and SCC*mec*, but also harbouring replicon types 20/21.

## Discussion

SARG is a newly described species within the SAUR complex and has been found distributed around the world causing a similar spectrum of human infections as SAUR. Phenotypic identification is difficult and remains the main challenge to many diagnostic laboratories, with reliance on colony appearance, biochemical tests such as Pyrrolidonyl aminopeptidase test, Staphylococcus Protein A, tube coagulase or MALDI-TOF MS mass spectrum profiles for identification [18]. The inclusion of SARG MSP into the Bruker database has made scientists aware of potential isolates, and mass peak profiles have been documented to differentiate the SAUR complex [9]. However, SARG still requires additional confirmation [9]. As no biochemical tests are available for definitive identification, molecular techniques such as nuclease (*nuc*) gene PCR are required [18,36, 37]. It is for this reason that the true incidence and disease spectrum of SARG has remained hidden due to misidentification as SAUR. Sanger sequencing of the nuclease gene is the most reliable means of identification, but this technique is not widely available in diagnostic laboratories. Likewise, applications using WGS are mostly limited to research or reference laboratories and not available to most diagnostic laboratories due to the cost and bioinformatic requirements.

SARG lack the dehydrosqualene synthase-encoding *ctrM*/*N*/*P*/*Q* genes required for the biosynthesis of staphyloxanthin, a pigment which provides oxidative protection from biological peroxides found in neutrophils and a major virulence factor in SAUR. WGS sequence analysis of our SARG strains showed no complete staphyloxanthin operon present, which is consistent with the selected global SARG sequences. Although SARG lacks this pathogenic feature, it is still a cause of disease in immunocompetent hosts with many other virulence genes associated with immune evasion, exotoxin production and cell adhesion.

A comparison of our NZ isolates with 175 global SARG genomes from the NCBI RefSeq nt database showed that many of the same SAUR virulence factors that are important for organism adaptation to different environments are also found in SARG. These virulence factors include type 8 polysaccharide capsule, host immune modification, haemolysis production (alpha and gamma), fibronectin-binding proteins, iron binding, immunoglobulin binding and proteinases. None of the NZ isolates had cytolytic toxin PVL genes *lukF/S* or *tsst-1* gene, but the analysis of the global sequences found seven isolates with *lukF/lukS*-PV and two isolates with *tsst-1* genes, indicating that they are rarely seen in SARG. Isolate 22CHL-3048L and seven global isolates (ST2793 clade) have a deletion of four *cap8* genes (*capH*, *capI*, *capJ* and *capK*), which are part of the operon encoding the capsular polysaccharide production and is a major pathogenic factor in SAUR. The capsular polysaccharide is important in phagocytosis evasion and is thought to help in antimicrobial resistance. The loss of *cap8* genes would have a detrimental effect on capsular production such as truncated or non-functional capsule production. Non-encapsulated SAUR isolated from bovine mastitis was not seen as less pathogenic but caused persistent infections [38]. This chronic mastitis was caused by the ability of non-encapsulated strains to adhere more effectively to host cells, become internalized and evade immune responses. Additionally, a study comparing acute and chronic osteomyelitis found a higher prevalence of non-encapsulated SAUR with chronic infection [39].

Antimicrobial resistance screening found that genes for fosfomycin, beta-lactams, trimethoprim and various efflux pump mechanisms are associated with fluoroquinolone or tetracycline resistance. Four out of the five NZ isolates possess the chromosomal *fosB* gene encoding fosfomycin resistance [32]. However, there are no EUCAST interpretive breakpoints for disc testing, but MICs are available for the confirmation of phenotypic resistance. The analysis of the 175 global strains found 152 (86.9%) isolates with the *fosB* gene. The *fosB* gene can be carried by mobile genetic elements such as plasmids where it is over-expressed [40] but is most commonly found on the chromosome [5]. The *fosB* gene has also been found in other *Staphylococcus* species such as *Staphylococcus capitis*, *S. warneri* and *Staphylococcus saprophyticus* [41].

Beta-lactam resistance was found to occur by two mechanisms: (i) penicillin resistance caused by class A beta-lactamase encoded by the *blaZ* gene and (ii) altered PBP2a encoded by the *mecA* gene. The *mecA* gene was found in isolates 19CHL-8664D, 20CHL-9983R and 22CHL-3048L, which all had SCC*mec* type IV 2Bc mobile genetic element. Isolates 19CHL-8664D and 20CHL-9983R were obtained from MRSA screening samples and 22CHL-3048L from blood culture; they were originally misidentified as MRSA. These isolates were phenotypically uncharacteristic of SAUR, with colony appearance lacking pigment on 5% SBA and altered colour on chromo-agar. Further investigation of the MALDI-TOF MSP indicated a possible match to SARG, prompting further analysis with WGS. Comparison with the 175 global sequences for beta-lactam resistance found *blaZ* enzyme is the most common mechanism of beta-lactam resistance with 111 (63.4%) out of 175 global isolates compared with 58 (33.1%) strains with *mecA*. Treatment of infections with methicillin-resistant SARG should be the same as for MRSA [18,42]. It was observed that the *dfrG* gene was only found in the SCC*mec*-containing isolates and 41/58 (70.7%) *mecA*-positive global isolates. No NZ isolates possessed the *tetL* gene, which is associated with tetracycline resistance, but it was found in 54/175 (30.9%) of global SARG isolates. No resistance mutations were detected in the *gyrA* or *parC* genes for fluoroquinolones or *ileS* gene for mupirocin, using the AMRFinder program. Also, a recent study described a SARG strain with the mupirocin resistance gene *mupA* encoded on a plasmid [43], but this was not detected in any of the five NZ strains or 175 global sequences obtained from the RefSeq database.

Plasmid analysis found near-identical or structurally similar plasmids in SAUR and SARG sequences in PLSDB. The *blaZ*-harbouring plasmids were ~20 kbp in size and exhibited the same gene synteny as global strains. This suggests that *blaZ* is mobilized on plasmids and transferred between *Staphylococcus* species [44]. Replicon typing of the *blaZ* plasmids was consistent between the NZ isolates and their matching PLSDB plasmids. The replicon types rep5a and rep16 were detected from the same plasmid, suggesting the presence of two replication initiator protein genes. However, this is highly unlikely, as such a rare occurrence would create conflicts in the replication machinery of the cell. Isolate 20CHL-2391H (*rep13*) contained a 4,788 bp plasmid fragment encoding a quaternary ammonium efflux pump (*qacC*). A PLSDB search found no matching plasmids, suggesting that this could be a novel plasmid. However, due to the limitations of short-read sequencing data, further plasmid analysis is difficult [45] and would require long-read sequencing (e.g. Nanopore MinION) data for accurate plasmid construct and gene feature characterization. Only isolate 21CHL-5842I did not contain a plasmid as determined by replicon typing, but the presence of an unidentified replicon type cannot be excluded and long-read sequencing would be needed to confirm.

A total of nine MLST types were identified among the 175 SARG sequences from RefSeq, with ST2250 (122/175) being the most common. The NZ isolates belong to MLST types ST2250 or ST2793. While MLST typing showed some correlation with the phylogenetic clades, its resolution power is limited due to the conserved nature of the selected genes. Core genome SNP-based analysis revealed that the NZ strains are distributed across different clades, suggesting strain diversity and multiple introductions via global dissemination of SARG. The phylogenetic tree from this study was comparable to the findings by Goswami *et al*. [5], which identified three distinct clades with five subclades. Clade A, the largest, contained three subclades that were predominately composed of MLST ST2250. The subclade A1 included the *dfrG* (trimethoprim resistance), *mecA* (beta-lactam resistance), which included 19CHL-8664D and 20CHL-9983R. Another notable clade is the clade C (ST2793), which contains isolate 22CHL-3084L. This clade consists of isolates lacking some of their *cap8* genes, but also harbouring *dfrG*, *mecA* and rep20 plasmid.

Four of the five NZ isolates were found from skin sites and one from a case of bacteraemia. The skin site isolates were all detected from MRSA screening samples, and two of the SARG isolates were methicillin resistant and indistinguishable from MRSA. The bloodstream isolate, which was methicillin resistant, underscores the pathogenic potential of this emerging organism and adds to the growing number of documented infections in the literature. The development of antimicrobial resistance due to the presence of the SCC*mec* element and plasmids, similar to SAUR, highlights the importance of accurately identifying and characterizing this unique species.

## Conclusion

In this article, we demonstrated the close relationship between SARG found in NZ and global strains, highlighting the worldwide spread of this emerging species. SARG is now recognized as a human pathogen, sharing many of the key virulence factors found in SAUR. The presence of the SCC*mec* and other antimicrobial resistance factors has important implications for treatment strategies, similar to those for SAUR. Since documented cases of SARG remain limited, further global investigations are needed to reduce sampling bias in the data and improve our understanding of this organism. This publication is the first documented occurrence of SARG in NZ.

## Abbreviations

EUCAST: European Committee on Antimicrobial Susceptibility Testing
MRSA: methicillin-resistant *Staphylococcus aureus*
MSP: mass spectral profiles
NZ: New Zealand
PBP: penicillin-binding protein
PLSDB: plasmid database
PVL: Panton–Valentine leukocidin
SARG: *Staphylococcus argenteus*
SAUR: *Staphylococcus aureus*
SBA: sheep blood agar
ST: sequence type

## References

[1] Ng JWS, Holt DC, Lilliebridge RA, Stephens AJ, Huygens F (2009). Phylogenetically distinct *Staphylococcus aureus* lineage prevalent among indigenous communities in northern Australia. J Clin Microbiol.

[2] Thaipadungpanit J, Amornchai P, Nickerson EK, Wongsuvan G, Wuthiekanun V (2015). Clinical and molecular epidemiology of *Staphylococcus argenteus* infections in Thailand. J Clin Microbiol.

[3] Hansen TA, Bartels MD, Høgh SV, Dons LE, Pedersen M (2017). Whole genome sequencing of Danish *Staphylococcus argenteus* reveals a genetically diverse collection with clear separation from *Staphylococcus aureus*. Front Microbiol.

[4] Witteveen S, Hendrickx APA, de Haan A, Notermans DW, Landman F (2022). Genetic characteristics of methicillin-resistant *Staphylococcus argenteus* isolates collected in the Dutch national MRSA surveillance from 2008 to 2021. Microbiol Spectr.

[5] Goswami C, Fox S, Holden M, Leanord A, Evans TJ (2021). Genomic analysis of global *Staphylococcus argenteus* strains reveals distinct lineages with differing virulence and antibiotic resistance gene content. Front Microbiol.

[6] Meijer EFJ, van Renssen A, Maat I, van der Graaf-van Bloois L, Duim B (2022). Canine *Staphylococcus argenteus*: case report from the Netherlands. Pathogens.

[7] Wakabayashi Y, Takemoto K, Iwasaki S, Yajima T, Kido A (2022). Isolation and characterization of *Staphylococcus argenteus* strains from retail foods and slaughterhouses in Japan. Int J Food Microbiol.

[8] Holt DC, Holden MTG, Tong SYC, Castillo-Ramirez S, Clarke L (2011). A very early-branching *Staphylococcus aureus* lineage lacking the carotenoid pigment staphyloxanthin. Genome Biol Evol.

[9] Schuster D, Rickmeyer J, Gajdiss M, Thye T, Lorenzen S (2017). Differentiation of *Staphylococcus argenteus* (formerly: *Staphylococcus aureus* clonal complex 75) by mass spectrometry from *S. aureus* using the first strain isolated from a wild African great ape. Int J Med Microbiol.

[10] Bogestam K, Vondracek M, Karlsson M, Fang H, Giske CG (2018). Introduction of a hydrolysis probe PCR assay for high-throughput screening of methicillin-resistant *Staphylococcus aureus* with the ability to include or exclude detection of *Staphylococcus argenteus*. PLoS One.

[11] Tunsjø HS, Kalyanasundaram S, Charnock C, Leegaard TM, Moen AEF (2018). Challenges in the identification of methicillin-resistant *Staphylococcus argenteus* by routine diagnostics. APMIS.

[12] Tong SYC, Schaumburg F, Ellington MJ, Corander J, Pichon B (2015). Novel staphylococcal species that form part of a *Staphylococcus aureus*-related complex: the non-pigmented *Staphylococcus argenteus* sp. nov. and the non-human primate-associated *Staphylococcus schweitzeri* sp. nov. Int J Syst Evol Microbiol.

[13] Chen S-Y, Lee H, Teng S-H, Wang X-M, Lee T-F (2018). Accurate differentiation of novel *Staphylococcus argenteus* from *Staphylococcus aureus* using MALDI-TOF MS. Future Microbiol.

[14] Chen S-Y, Lee H, Wang X-M, Lee T-F, Liao C-H (2018). High mortality impact of *Staphylococcus argenteus* on patients with community-onset staphylococcal bacteraemia. Int J Antimicrob Agents.

[15] Rigaill J, Grattard F, Grange S, Forest F, Haddad E (2018). Community-acquired *Staphylococcus argenteus* sequence type 2250 bone and joint infection, France, 2017. Emerg Infect Dis.

[16] Chantratita N, Wikraiphat C, Tandhavanant S, Wongsuvan G, Ariyaprasert P (2016). Comparison of community-onset *Staphylococcus argenteus* and *Staphylococcus aureus* sepsis in Thailand: a prospective multicentre observational study. Clin Microbiol Infect.

[17] Aung MS, San T, Aye MM, Mya S, Maw WW (2017). Prevalence and genetic characteristics of *Staphylococcus aureus* and *Staphylococcus argenteus* isolates harboring Panton-Valentine leukocidin, enterotoxins, and TSST-1 genes from food handlers in Myanmar. Toxins.

[18] Eshaghi A, Bommersbach C, Zittermann S, Burnham CA, Patel R (2021). Phenotypic and genomic profiling of *Staphylococcus argenteus* in Canada and the United States and recommendations for clinical result reporting. J Clin Microbiol.

[19] Mores CR, Montelongo C, Putonti C, Wolfe AJ, Abouelfetouh A (2021). Investigation of plasmids among clinical *Staphylococcus aureus* and *Staphylococcus haemolyticus* isolates from Egypt. Front Microbiol.

[20] Neyaz L, Rajagopal N, Wells H, Fakhr MK (2020). Molecular characterization of *Staphylococcus aureus* plasmids associated with strains isolated from various retail meats. Front Microbiol.

[21] Firth N, Jensen SO, Kwong SM, Skurray RA, Ramsay JP (2018). Staphylococcal plasmids, transposable and integrative elements. Microbiol Spectr.

[22] Steward CD, Raney PM, Morrell AK, Williams PP, McDougal LK (2005). Testing for induction of clindamycin resistance in erythromycin-resistant isolates of *Staphylococcus aureus*. J Clin Microbiol.

[23] Chklovski A, Parks DH, Woodcroft BJ, Tyson GW (2023). CheckM2: a rapid, scalable and accurate tool for assessing microbial genome quality using machine learning. Nat Methods.

[24] Wood DE, Lu J, Langmead B (2019). Improved metagenomic analysis with Kraken 2. Genome Biol.

[25] Feldgarden M, Brover V, Haft DH, Prasad AB, Slotta DJ (2019). Validating the AMRFinder tool and resistance gene database by using antimicrobial resistance genotype-phenotype correlations in a collection of isolates. Antimicrob Agents Chemother.

[26] Seemann T (2014). Prokka: rapid prokaryotic genome annotation. Bioinformatics.

[27] Tonkin-Hill G, MacAlasdair N, Ruis C, Weimann A, Horesh G (2020). Producing polished prokaryotic pangenomes with the Panaroo pipeline. Genome Biol.

[28] Croucher NJ, Page AJ, Connor TR, Delaney AJ, Keane JA (2015). Rapid phylogenetic analysis of large samples of recombinant bacterial whole genome sequences using Gubbins. Nucleic Acids Res.

[29] Minh BQ, Schmidt HA, Chernomor O, Schrempf D, Woodhams MD (2020). IQ-TREE 2: new models and efficient methods for phylogenetic inference in the genomic era. Mol Biol Evol.

[30] Schwengers O, Jelonek L, Dieckmann MA, Beyvers S, Blom J (2021). Bakta: rapid and standardized annotation of bacterial genomes via alignment-free sequence identification. Microb Genom.

[31] Prjibelski A, Antipov D, Meleshko D, Lapidus A, Korobeynikov A (2020). Using SPAdes de novo assembler. Curr Protoc Bioinformatics.

[32] Fu Z, Liu Y, Chen C, Guo Y, Ma Y (2016). Characterization of fosfomycin resistance gene, *fosB*, in methicillin-resistant *Staphylococcus aureus* isolates. PLoS One.

[33] Schmartz GP, Hartung A, Hirsch P, Kern F, Fehlmann T (2022). PLSDB: advancing a comprehensive database of bacterial plasmids. Nucleic Acids Res.

[34] McCarthy AJ, Lindsay JA (2012). The distribution of plasmids that carry virulence and resistance genes in *Staphylococcus aureus* is lineage associated. BMC Microbiol.

[35] Amirsoleimani A, Brion G, Francois P (2021). Co-carriage of metal and antibiotic resistance genes in sewage-associated staphylococci. Genes.

[36] Kaden R, Engstrand L, Rautelin H, Johansson C (2018). Which methods are appropriate for the detection of *Staphylococcus argenteus* and is it worthwhile to distinguish *S. argenteus* from *S. aureus*?. Infect Drug Resist.

[37] Supriadi IR, Santosaningsih D, Budayanti NS, Zandijk WHA, Rijfkogel A (2024). Identification and characterization of *Staphylococcus argenteus* from Indonesia. Int J Med Microbiol.

[38] Rossi BF, Bonsaglia ÉCR, Castilho IG, Dantas STA, Langoni H (2020). First investigation of *Staphylococcus argenteus* in a Brazilian collections of *S. aureus* isolated from bovine mastitis. BMC Vet Res.

[39] Lattar SM, Tuchscherr LPN, Caccuri RL, Centrón D, Becker K (2009). Capsule expression and genotypic differences among *Staphylococcus aureus* isolates from patients with chronic or acute osteomyelitis. Infect Immun.

[40] Etienne J, Gerbaud G, Fleurette J, Courvalin P (1991). Characterization of staphylococcal plasmids hybridizing with the fosfomycin resistance gene fosB. FEMS Microbiol Lett.

[41] Osada M, Aung MS, Urushibara N, Kawaguchiya M, Ohashi N (2022). Prevalence and antimicrobial resistance of *Staphylococcus aureus* and coagulase-negative *Staphylococcus*/ *Mammaliicoccus* from retail ground meat: identification of broad genetic diversity in fosfomycin resistance gene *fosB*. Pathogens.

[42] Becker K, Schaumburg F, Kearns A, Larsen AR, Lindsay JA (2019). Implications of identifying the recently defined members of the *Staphylococcus aureus* complex *S. argenteus* and *S. schweitzeri*: a position paper of members of the ESCMID Study Group for Staphylococci and Staphylococcal Diseases (ESGS). Clin Microbiol Infect.

[43] Shittu AO, Layer-Nicolaou F, Strommenger B, Nguyen M-T, Bletz S (2022). First report of a methicillin-resistant, high-level mupirocin-resistant *Staphylococcus argenteus*. Front Cell Infect Microbiol.

[44] Olsen JE, Christensen H, Aarestrup FM (2006). Diversity and evolution of blaZ from *Staphylococcus aureus* and coagulase-negative staphylococci. J Antimicrob Chemother.

[45] Arredondo-Alonso S, Willems RJ, van Schaik W, Schürch AC (2017). On the (im)possibility of reconstructing plasmids from whole-genome short-read sequencing data. Microb Genom.

